# The Mutation Spectrum of Maturity Onset Diabetes of the Young (MODY)-Associated Genes among Western Siberia Patients

**DOI:** 10.3390/jpm11010057

**Published:** 2021-01-18

**Authors:** Dinara E. Ivanoshchuk, Elena V. Shakhtshneider, Oksana D. Rymar, Alla K. Ovsyannikova, Svetlana V. Mikhailova, Veniamin S. Fishman, Emil S. Valeev, Pavel S. Orlov, Mikhail I. Voevoda

**Affiliations:** 1 Federal Research Center Institute of Cytology and Genetics, Siberian Branch of Russian Academy of Sciences (SB RAS), Prospekt Lavrentyeva 10, 630090 Novosibirsk, Russia; shakhtshneyderev@bionet.nsc.ru (E.V.S.); mikhail@bionet.nsc.ru (S.V.M.); minja@bionet.nsc.ru (V.S.F.); emil@bionet.nsc.ru (E.S.V.); orlovpavel86@gmail.com (P.S.O.); mvoevoda@ya.ru (M.I.V.); 2 Institute of Internal and Preventive Medicine—Branch of Institute of Cytology and Genetics, SB RAS, Bogatkova Str. 175/1, 630004 Novosibirsk, Russia; orymar23@gmail.com (O.D.R.); aknikolaeva@bk.ru (A.K.O.)

**Keywords:** maturity onset diabetes of the young, MODY, diabetes mellitus, multiplex ligation-dependent probe amplification, next-generation sequencing, GCK, HNF1A, HNF4A, HNF1B, single-nucleotide variant, population

## Abstract

Maturity onset diabetes of the young (MODY) is a congenital form of diabetes characterized by onset at a young age and a primary defect in pancreatic-β-cell function. Currently, 14 subtypes of MODY are known, and each is associated with mutations in a specific gene: *HNF4A*, *GCK*, *HNF1A*, *PDX1*, *HNF1B*, *NEUROD1*, *KLF11*, *CEL*, *PAX4*, *INS*, *BLK*, *KCNJ11*, *ABCC8*, and *APPL1*. The most common subtypes of MODY are associated with mutations in the genes *GCK*, *HNF1A*, *HNF4A*, and *HNF1B*. Among them, up to 70% of cases are caused by mutations in *GCK* and *HNF1A*. Here, an analysis of 14 MODY genes was performed in 178 patients with a MODY phenotype in Western Siberia. Multiplex ligation-dependent probe amplification analysis of DNA samples from 50 randomly selected patients without detectable mutations did not reveal large rearrangements in the MODY genes. In 38 patients (37% males) among the 178 subjects, mutations were identified in *HNF4A*, *GCK*, *HNF1A*, and *ABCC8*. We identified novel potentially causative mutations p.Lys142*, Leu146Val, Ala173Glnfs*30, Val181Asp, Gly261Ala, IVS7 c.864 −1G>T, Cys371*, and Glu443Lys in *GCK* and Ser6Arg, IVS 2 c.526 +1 G>T, IVS3 c.713 +2 T>A, and Arg238Lys in *HNF1A*.

## 1. Introduction

Maturity onset diabetes of the young (MODY) is a congenital form of diabetes characterized by onset at a young age and a primary defect in pancreatic-β-cell function. This type of diabetes (OMIM # 606391) differs from classic types of diabetes mellitus (types 1 and 2, or T1DM and T2DM) in its clinical course, treatment strategies, and prognosis [1,2]. MODY is predominantly inherited in an autosomal dominant manner, but cases of spontaneous mutagenesis and germline mosaicism have been described [3,4]. MODY can be suspected [5] when hyperglycemia is detected in patients under 25–35 years of age, there is little or no need for insulin, secretion of C-peptide is intact, β-cell antibodies are absent, and dysfunction of pancreatic β-cells results in a decrease in the insulin amount. MODY patients usually have a normal body mass index. Currently, 14 subtypes of MODY are known, and each is associated with mutations in a specific gene: *HNF4A*, *GCK*, *HNF1A*, *PDX1*, *HNF1B*, *NEUROD1*, *KLF11*, *CEL*, *PAX4*, *INS*, *BLK*, *KCNJ11*, *ABCC8*, and *APPL1* [6]. The genes associated with MODY are presented in Table 1.

The most common subtypes of MODY are associated with mutations in the genes *GCK*, *HNF1A*, *HNF4A*, and *HNF1B* [7]; among them, up to 70% of cases are caused by mutations in *GCK* and *HNF1A* [8]. Clinical manifestations of MODY are diverse and may vary even among members of the same family, i.e., carriers of identical mutations. This phenotypic variation is due to the interaction of the mutations with different genetic backgrounds and with environmental factors (e.g., the lifestyle) [9]. Identification of mutations causing MODY is important for early diagnosis of the disease in first-degree relatives of a proband. Up to 80% of MODY cases remain undetected or are misdiagnosed as T1DM or T2DM, resulting in incorrect treatment, including unjustified insulin therapy and its complications [1]. Next-generation sequencing (NGS) is most commonly used in MODY diagnostics and allows for simultaneous analysis of numerous genes and for the identification of single-nucleotide variants (SNVs) or small deletions or insertions, which constitute the majority of the known mutations causing this disease [10]. Nonetheless, large rearrangements in MODY-associated genes have been described, including heterozygosity of a complete *HNF1B* deletion [11,12], detected by multiplex ligation-dependent probe amplification (MLPA). This method makes it possible to detect variations in the copy number of a gene and accordingly is widely used in molecular diagnostics of diseases whose pathogenesis is associated with deletions or duplications of certain genomic regions [13]. The purpose of the present study was to determine the spectrum of genetic variants in patients with a MODY phenotype in Russia (Western Siberia) by whole-exome sequencing, targeted sequencing, and MLPA.

## 2. Materials and Methods

### 2.1. Patients

The study protocol was approved by the Ethics Committee of the Institute of Internal and Preventive Medicine- branch of the Institute of Cytology and Genetics, SB RAS, Novosibirsk, Russia, protocol number 7, 22 June 2008. Written informed consent to be examined and to participate in the study was obtained from each patient or his/her parent or legal guardian.

**Figure 1:**
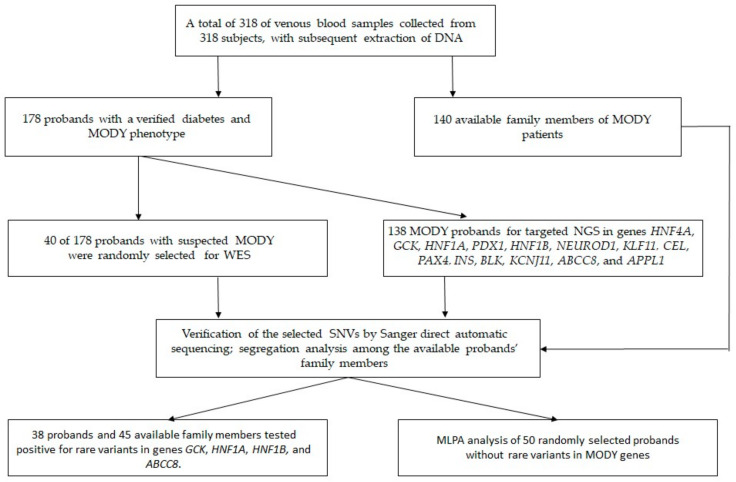
The study design. DNA: deoxyribonucleic acid, MLPA: multiplex ligation-dependent probe amplification, MODY: maturity onset diabetes of the young, SNV: single-nucleotide variant, WES: whole-exome sequencing.

A total of 178 unrelated patients aged 4 to 35 years (23.4 ± 11.1 years [mean ± SD]; 41.7% males) with a MODY phenotype and 140 available family members were enrolled in this study (Figure 1).

All 178 probands were examined in the Clinical Department of the Institute of Internal and Preventive Medicine from the year 2014 to 2020. The probands were referred for molecular genetic testing if they met the following criteria: a verified diagnosis of diabetes [according to the criteria of the American Diabetes Association: HbA1C ≥6.5%, or fasting plasma glucose ≥126 mg/dL (7.0 mmol/L), or 2-h plasma glucose ≥200 mg/dL (11.1 mmol/L) during an oral glucose tolerance test (in the absence of unequivocal hyperglycemia; the result should be confirmed by repeat testing), or a patient with classic symptoms of hyperglycemia or hyperglycemic crisis with a random plasma glucose ≥200 mg/dL (11.1 mmol/L)] [14]; a debut of the disease in probands at the age of 35 years or earlier; a family history of diabetes mellitus; the absence of obesity; the absence of antibodies against pancreas islet cells and glutamic acid decarboxylase; intact secretory function of β-cells; normal or mildly reduced C-peptide levels; no need for insulin therapy; and the absence of ketoacidosis at the onset of the disease. Patients with clinical features of untypical diabetes (differing from those of T1DM and T2DM) and, in some cases, lacking a family history were included in this study because MODY can be occasionally caused by de novo mutations [3,4]. Patients with a history of tuberculosis, infection with the human immunodeficiency virus, an infectious disease caused by hepatitis B or C virus that required antiviral treatment, or substance abuse or alcoholism in the 2 years before the examination were excluded from this study. The studied MODY group may have included patients with MODY, patients with T1DM and negative test results on antibodies, and patients with early-onset T2DM.

### 2.2. Isolation of Genomic DNA

Genomic DNA was isolated from venous blood leukocytes by phenol–chloroform extraction [15].

### 2.3. Genome Library Preparation and Sequencing

At the first stage, exome sequencing was performed on 40 randomly selected probands from the MODY group. The exome sequencing was carried out on an Illumina HiSeq 1500 instrument (Illumina, San Diego, CA, USA). The enrichment and library preparation were performed using the SureSelectXT Human All Exon v.5 + UTRs Kit. Whole-exome libraries were prepared with the AmpliSeq Exome Kit (Thermo Fisher Scientific, Walthamm, MA, USA). On the DNA samples from the other patients (138 unrelated probands), we performed targeted sequencing. In the target panel, we included coding parts and adjacent splicing sites of the following MODY-associated genes: *HNF4A*, *GCK*, *HNF1A*, *PDX1*, *HNF1B*, *NEUROD1*, *KLF11*, *CEL*, *PAX4*, *INS*, *BLK*, *KCNJ11*, *ABCC8*, and *APPL1*. Oligodeoxynucleotide probes and the KAPA HyperPlus Kit (Roche, Basel, Switzerland) were used to prepare libraries at the Genomics Multi-Access Center (Institute of Cytology and Genetics, SB RAS, Novosibirsk, Russia). The quality of the analyzed DNA and of the prepared libraries was evaluated by means of a capillary electrophoresis system, the Agilent 2100 Bioanalyzer (Agilent Technologies Inc., Santa Clara, CA, USA). Analysis of each fully prepared library was conducted on the Illumina MiSeq platform (Illumina, San Diego, CA, USA).

### 2.4. Bioinformatic Analysis

The sequence reads were mapped to the reference human genome (GRCh37/hg19) via the Burrow–Wheeler Alignment tool (BWA v.0.7.17) [16]. Polymerase chain reaction (PCR)-generated duplicates were removed with MarkDuplicates of PicardTools (https://gatk.broadinstitute.org/hc/en-us). Base quality recalibration and searches for SNVs were conducted using Genome Analysis Toolkit (GATK) v.3.3, with BaseRecalibration and HaplotypeCaller tools, respectively; variant calling included local remapping of short insertions/deletions and correction of base quality [17].

The depth of coverage was 34× to 53×. SNVs with genotype quality scores <20 were filtered out and excluded from further analysis. In sequenced groups, we kept sequence variants if they were present in 10 or more variant reads with a quality score ≥20. Annotation of the SNVs was performed in the ANNOVAR (ANNOtate VARiation) software [18] with the help of populational data gnomAD [19] as a basic source and databases for several specific populations, such as The Greater Middle East (GME) Variome Project (http://igm.ucsd.edu/gme/), ABraOM: Brazilian genomic variants (http://abraom.ib.usp.br/), Korean Personal Genome Project (http://opengenome.net/Main_Page), other available data from populations and data on clinical significance (ClinVar) [20], and the Human Gene Mutation Database (HGMD) [10]; literature data were taken into account too.

Possible functional effects of SNVs were assessed in the dbNSFP database (https://sites.google.com/site/jpopgen/dbNSFP), aggregating data from 37 in silico prediction tools (SIFT, Polyphen2, MutationTaster2, PROVEAN, and others), nine conservation scores (e.g., PhyloP, phastCons, GERP++, and SiPhy), and data on population frequencies. Thresholds for prediction scores were set according to respective authors’ recommendations; for the conservation ratio, we set one single threshold at 0.7, which means that a variant can be considered conserved if its predicted conservation score is greater than the scores of 70% variants.

Variants described in ClinVar, Leiden Open Variation Database (https://www.lovd.nl/), or predicted in silico as benign (B) or likely benign (LB) as well as variants with minor allele frequency higher than 0.01% in any of the population databases listed above were excluded from the analysis. The pathogenicity of each novel candidate mutation was assessed according to the recommendations of the American College of Medical Genetics and Genomics (ACMG) and the Association for Molecular Pathology [21]. Using these criteria, we also assessed two variants (p.Val590Met in the *HNF1A* gene in proband P81 and p.Ala173Thr in the *GCK* gene in P90) previously reported without any description of a phenotype (Table 2).

To identify pathogenic variants at potential splice sites, we performed in silico testing, using dbscSNV (http://www.liulab.science/dbscsnv.html) for splice sites and regSNP-intron (https://regsnps-intron.ccbb.iupui.edu/) for both intronic and splice-altering SNVs. Nevertheless, these databases can be only a supplementary tool for the estimation of variant pathogenicity, in addition to the population and clinical data.

### 2.5. Verification Analysis

All selected SNVs were verified by Sanger direct automatic sequencing on an ABI 3500 DNA sequencer (Thermo Fisher Scientific, USA) by means of the BigDye Terminator v3.1 Cycle Sequencing Kit (Thermo Fisher Scientific, USA). Primer design for the selected SNVs was performed in the Primer-Blast software (https://www.ncbi.nlm.nih.gov/tools/primer-blast/).

At the second stage, segregation analysis was performed on the selected variants (novel and previously described) in the probands’ family members available for the analysis.

### 2.6. MLPA Analysis

The third stage included an MLPA analysis of DNA samples from 50 randomly selected patients without detectable mutations in MODY-associated genes to search for major structural rearrangements (deletions and duplications). MLPA was performed using SALSA MLPA Probemix P241 MODY Mix 1 (MRC-Holland, Amsterdam, Netherlands), containing 52 specific MLPA probes for four genes: *HNF4A*, *GCK*, *HNF1A*, and *HNF1B* (https://www.mrcholland.com/product/P241/2528). The reaction was carried out in accordance with the manufacturer’s recommendations. The resulting amplification products were detected by capillary electrophoresis on an ABI 3500 DNA sequencer (Thermo Fisher Scientific, USA). The data were processed in the Coffalyser.Net software.

## 3. Results

In 40 probands with a MODY phenotype, whole-exome analysis was performed; in 138 probands, we analyzed exons and adjacent splice sites of MODY-associated genes: *HNF4A*, *GCK*, *HNF1A*, *PDX1*, *HNF1B*, *NEUROD1*, *KLF11*, *CEL*, *PAX4*, *INS*, *BLK*, *KCNJ11*, *ABCC8*, and *APPL1*. In this work, we ignored common genetic variants and did not assess their possible effects on the phenotype. The results are presented in Table 2. We did not find any rare pathogenic variants in genes *HNF4A*, *PDX1*, *NEUROD1*, *KLF11*, *CEL*, *PAX4*, *INS*, *BLK*, *KCNJ11*, and *APPL1*. In 38 patients (37% males) out of the 178 subjects, mutations were identified in *HNF4A*, *GCK*, *HNF1A*, and *ABCC8* (one in a compound heterozygous state and 37 in a heterozygous state). The vast majority of them were carriers of rare variants of the *GCK* gene (26 probands; 68.4%), and nine probands (26.3%) had mutations in the *HNF1A* gene; one male (2.6%) carried a pathogenic variant in the *ABCC8* gene, and one female was a carrier of a rare variant in *HNF1B*. One male patient (Р73) was a compound heterozygote on two previously described mutations: Arg54* in *HNF1A* and Arg521Gln in *ABCC8*.

In total, we identified 36 rare variants in the studied genes: 12 of them (33.3%) are described for the first time, and the rest are present in databases (LOVD, HGMD, ClinVar, and/or gnomAD) or are previously reported in the literature. Among the identified variants, only three (all located in the *GCK* gene) were found twice in unrelated patients. Probands Р3 and Р77 both were found to carry Gly258Cys, probands Р46 and Р80 to carry undescribed Val181Asp, and probands Р6 and Р50 to carry Arg36Trp in the *GCK* gene. All the novel variants proved to be likely pathogenic or pathogenic according to the ACMG criteria [21]. For 28 families with identified relevant variants, a segregation analysis was performed. For most patients, segregation of the identified mutations with pathogenic phenotypes was found among their relatives.

An exception was the His336Asp mutation in *HNF1B* in a patient (P27) with gestational diabetes. The same mutation was found in her normoglycemic mother and her daughter.

In 50 patients without mutations in the studied genes, the MLPA analysis did not reveal any structural abnormalities in the genes *HNF4A*, *GCK*, *HNF1A*, and *HNF1B*.

## 4. Discussion

**Figure 2:**
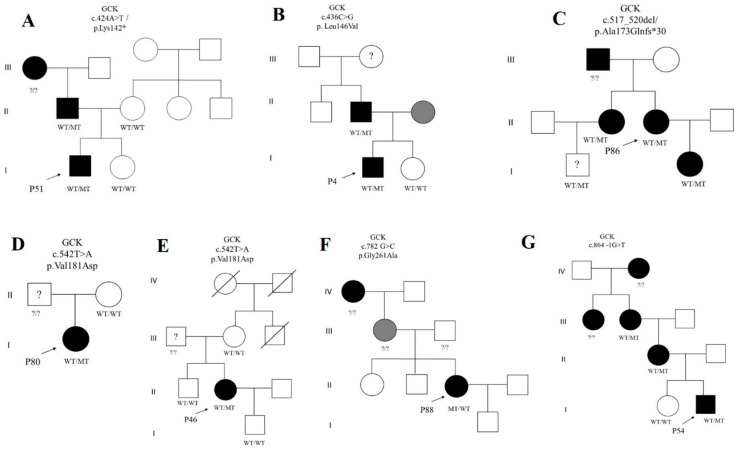

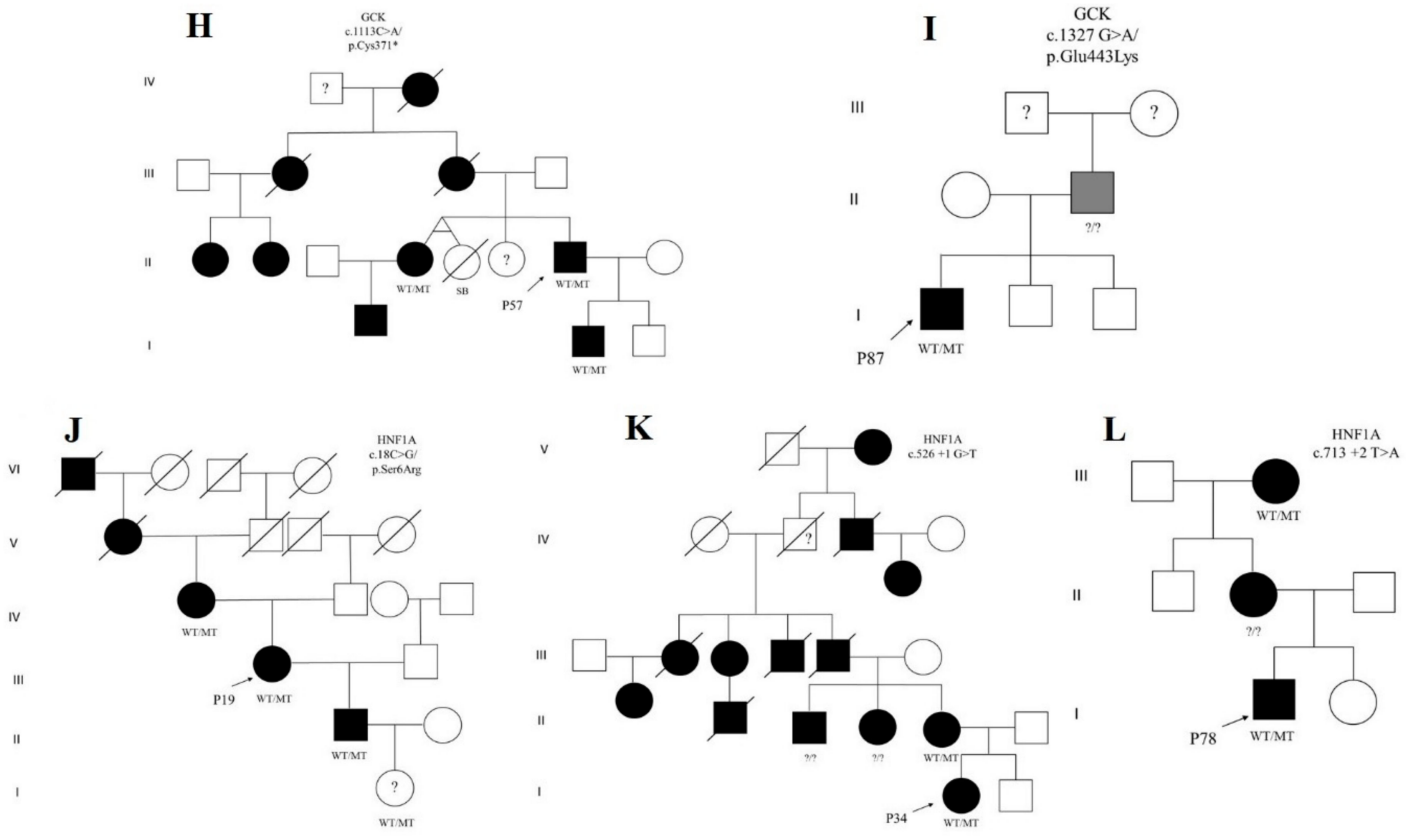
Screened MODY families (**A**–**L**) with novel identified variants in genes *GCK* and *HNF1A*. A grey filled symbol: a patient with prediabetes, MT: altered allele, SB: stillbirth, WT: wild type allele, ?/?: persons not genotyped, ?: a person with unknown health status. * RefSeq reference transcript: GCK (NM_000162.5), ABCC8 (NM_000352.3), HNF1B (NM_000458.2), and HNF1A (NM_000545.6).

Genetic diagnosis is an important step in clinical practice, especially for family screening of individuals with borderline or moderate carbohydrate metabolic disorders. Clinical manifestations of MODY differ among patients and do not allow us to identify the type of diabetes unambiguously; therefore, it is important to employ timely genetic diagnostics for the optimal choice of a treatment. In the present study, we searched for mutations in MODY-associated genes by combining an examination of clinical criteria with NGS (the latter method has been effectively used as a first-line screening test for MODY-associated mutations). Next, the identified mutations were verified by Sanger sequencing followed by cascade genetic screening of available family members. In a Siberian population, we have previously reported some of the mutations: c.580 –1G>A in *GCK* [23], Ser6Arg in *HNF1A* [24], and Ala1457Thr in *ABCC8* [25]. Among the studied patients (all from Western Siberia), the predominance of subtypes MODY2 (68.4%) and MODY3 (26.3%) was demonstrated here. We found eight novel genetic variants in *GCK* and four novel variants in *HNF1A* in this work (Figure 2, Table 2).

Routine or accidental blood glucose testing is the main route of hyperglycemia detection in *GCK*-MODY. The disease is characterized by an insignificant increase in the fasting glucose level (well controlled without medication) and low prevalence of micro- and macrovascular complications of diabetes [2]. Encoded by the *GCK* gene, glucokinase B (GlkB, hexokinase IV) is the first enzyme in the glycolytic pathway and phosphorylates glucose in pancreatic β-cells [26].

The crystal structure of human glucokinase indicates that it has a large domain and small domain forming a deep cleft for glucose binding [27]. Amino acid residues 1–64 and 206–439 belong to the large domain, and residues 72–201 and 445–465 belong to the small domain; residues 65–71, 202–205, and 440–444 constitute three loops connecting the domains. Glucokinase is switched from an inactive to active conformation by ligand binding via a large rotation of the small domain [27,28]. Many mutations of this gene have been described (nonsense, missense, frameshift, and splice site mutations) in various populations [10]. Of note, all *GCK*-MODY patients have similar clinical phenotypes regardless of the mutation type [29].

p.Lys142* was found here in a 10-year-old boy (P51 in family A) with hyperglycemia. Lysine at codon 142 is adjacent to three basic amino acid residues (His-141, Lys-142, and Lys-143) that are phylogenetically conserved and form a positively charged surface. This basic patch in GlkB is believed to be a critical site for the binding of glucokinase-regulatory protein because substitutions at this site decrease this binding and nuclear entry of GlkB [30]. There are no reports on mutations in this codon in the literature.

The Leu146Val substitution is located in the small domain and was found in a 12-year-old male proband here (P4 in family B). Within codon 146, two other variants (Leu146Pro and Leu146Arg) have been described, both associated with MODY [10]. Research on enzyme kinetics has revealed that mutation Leu146Arg reduces enzymatic activity owing to decreased affinity for glucose [31].

A novel frameshift mutation, p.Ala173Glnfs*30, was identified here in family C with a MODY2 phenotype. Namely, this heterozygous deletion of four nucleotides in exon 5 was detected in a young woman 35 years of age (P86), in her son, and in her sibling. This AGGC deletion in codon 173 of the *GCK* gene changes the amino acid sequence and generates a premature stop codon at amino acid position 203. In our study, this variant segregated with the pathological phenotype in the proband and proband’s daughter and sister. In view of the young age of the proband’s nephew, monitoring of his carbohydrate metabolism parameters was recommended. Enzyme kinetics should be assessed to determine the functional basis of the disease.

In two unrelated female patients (P80 in family D and P46 in family E), a variant was found resulting in the replacement of valine in codon 181 with aspartic acid. In both families, the supposed carriers (fathers) of the Val181Ala variant were not available for the analysis; it turned out that healthy mothers of the probands and relatives of P46 are not carriers of this substitution. Val181Ala in a family of a MODY patient was described in Italy [32]. In the present study, a young patient (P88 in family F) with gestational diabetes mellitus and a family history of diabetes was found to have a previously undescribed missense substitution, Gly261Ala, in the *GCK* gene. Earlier, two substitution variants of this codon—Gly261Arg and Gly261Glu—were found in France [33,34], both associated with MODY.

Here, in a 2-year-old boy (P54 in family G) with hyperglycemia, the c.864 −1G>T substitution was identified in the acceptor site of *GCK* intron 7. This variant segregated with a pathological phenotype in the examined family members. Substitutions −1G>A and −1G>C at this position have been described in the United Kingdom and Czech Republic [35,36].

Several variants in codon Cys371 have been described [10]. We first identified a nonsense variant in this codon in patient P57 (family H). The substitution segregated with a pathological phenotype in other family members. Cys371 is a highly conserved amino acid residue located in the hydrophobic core of the protein [37], where it participates in disulfide bond formation [38].

It is known that l-arginine stimulates the production of glucose-6-phosphate and induces insulin secretion [39]. In a male patient (P87) in family I, the p.Glu443Lys variant was detected, which is involved in l-arginine binding and insulin secretion [39]. Glu443 is located in the α-13 helix of human GlkB [26]. During the domain reorganization between the active and inactive forms of the enzyme, helices α-13 and α-5 take part in the global conformational change [27].

Hepatocyte nuclear factor I homeobox A (HNF1A) is a transcription factor regulating the differentiation of pancreatic cells [40]. Mutations in the *HNF1A* gene are associated with MODY3, which is characterized by impaired insulin secretion, retention of sensitivity to sulfonylureas, and a decrease in the renal threshold for glucose. Cases of liver adenomatosis, renal dysplasia, and hypopituitarism in carriers of these mutations have been documented [41,42]. The clinical manifestations of MODY3 can vary within the same family and among unrelated mutation carriers. Moreover, carriers of *HNF1A* gene mutations may be normoglycemic, while their siblings can be hyperglycemic [43].

The Ser6Arg mutation of the *HNF1A* gene was found here in a family with diabetes mellitus in five generations [24] in proband P19 (family J). Another variant, c.526 +1 G>T in intron 2 of *HNF1A*, was detected in a 12-year-old patient (proband P34 in family K). She was found to have fasting hyperglycemia of 11.3 mmol/L and glycosuria of more than 55 mmol/L. In the proband’s family, this variant segregated with a pathological phenotype and has not been described previously.

Proband P91 is a carrier of novel variant Arg238Lys. Two other substitutions at the same position, Arg238Met and Arg238Thr, have been previously described in the LOVD database as likely pathogenic (HNF1A_000306 and HNF1A_000417). One of them, Arg238Thr, is associated with MODY in the United Kingdom [44], and the other one, Arg238Met, is associated with hyperinsulinism diagnosed at birth (LOVD database). According to the proband, her ancestors had diabetes mellitus for three generations, but they were not available for analysis.

One of our young male patients (Р78 in family L) has substitution c.713 +2 T>A in intron 3 of the *HNF1A* gene. In an available relative of the proband, this mutation segregated with non-insulin-dependent diabetes mellitus and was found for the first time.

ATP-binding cassette superfamily transporter family C8 gene (*ABCC8*) encodes sulfonylurea receptor 1 (SUR1), which is a part of ATP-sensitive potassium channels on the islet β-cell membrane. Mutations in this gene can cause T2DM [45], a transient type of neonatal diabetes [46], neonatal diabetes [47], or MODY12 [6]. Previously, for the first time in Russia, we described the case of a patient with an *ABCC8* mutation [25]. In the current study, a 13-year-old male patient (Р73, family history is not shown) proved to be compound heterozygous on Arg521Gln of *ABCC8* and previously reported Arg54* of *HNF1A*. His mother, also with diabetes mellitus, has the same genotype. Elsewhere, the Arg521Gln substitution in the ABCC8 protein has been identified in a female with dominant hyperinsulinism [48] and in a case of a heterozygous carrier with diabetes [49]. In our case, neither the mother nor her son had any relevant complications.

A mutation in the *HNF1B* gene results in MODY5, which is characterized by reduced insulin secretion and usually a renal disease [50]. This gene plays the major part in the normal development of (and tissue-specific gene expression in) the kidneys, liver, pancreas, bile ducts, urogenital tract, lungs, thymus, and gut [50]. In patient P27 (family history is not shown), a diagnosis of gestational diabetes was made. Mutation His336Asp in *HNF1B* was detected in a proband and in her normoglycemic mother and daughter; no other *HNF1B*-associated clinical phenotypes, such as genital deformities or kidney involvement, were revealed in this proband [51]. The information on the pathogenicity of this variant is unclear. This mutation has been identified and categorized as pathogenic in two unrelated patients with severe renal anomalies, but healthy relatives of the patients are reported to be carriers of the mutation as well [52]. In another study, two out of three affected members from one family were carriers of this mutation, and it was found in one healthy individual [53]. In other studies, this variant was identified in a patient with suspected T1DM and absence of autoimmunity, but information about their parents was not available [54]. It is likely that the mutation does not have 100% penetrance and that other modulating pathological factors, including genetic ones, are required for disease manifestation.

In Russia, genetic screening of patients with MODY has been previously performed in the European part of Russia, and mutations in the *GCK* gene seem to be most prevalent among these MODY patients [55,56].

Direct sequencing of MODY genes is time-consuming and does not cover the entire spectrum of genes that can cause this type of diabetes. NGS techniques enable more efficient and cost-effective diagnosis of MODY subtypes [57]. High-tech sequencing also helps to determine the cause of a MODY phenotype if mutations in the genes known to be associated with MODY1–MODY14 are absent.

### Limitations

This study has some limitations due to the unavailability of information about probands’ family members (in some cases). This situation did not allow us to perform a segregation analysis of some rare potentially pathogenic variants identified in our patients (data not shown).

## 5. Conclusions

The spectrum of mutations in MODY genes was determined in a Western Siberian population by NGS. We believe that NGS techniques will lead to more effective and cost-efficient methods of MODY diagnosis. Apparently, mutations in the *GCK* gene are the predominant cause of MODY in Russia. We identified novel potentially causative mutations p.Lys142*, Leu146Val, Ala173Glnfs*30, Val181Asp, Gly261Ala, IVS7 c.864 −1G>T, Cys371*, and Glu443Lys in *GCK* and Ser6Arg, IVS 2 c.526 +1 G>T, IVS3 c.713 +2 T>A, and Arg238Lys in *HNF1A* (both are known MODY-associated genes). We did not find large rearrangements in MODY genes in a randomly selected cohort of 50 patients devoid of relevant point mutations.

## Figures and Tables

**Table 1:** The genes associated with maturity onset diabetes of the young (MODY).

MODY Type	Gene	Protein Name	Genomic Location GRCh37 (hg19)
MODY1	*HNF4A*	Hepatic Nuclear Factor 4 Alpha	chr20:42,984,340-43,061,485
MODY2	*GCK*	Glucokinase (Hexokinase 4)	chr7:44,183,870-44,237,769
MODY3	*HNF1A*	Hepatocyte Nuclear Factor 1-Alpha	chr12:121,416,346-121,440,315
MODY4	*PDX1*	Pancreatic and Duodenal Homeobox 1	chr13:28,494,157-28,500,451
MODY5	*HNF1B*	Hepatocyte Nuclear Factor 1-Beta	chr17:36,046,434-36,105,237
MODY6	*NEUROD1*	Neuronal Differentiation 1	chr2:182,537,815-182,545,603
MODY7	*KLF11*	Krüppel-Like Factor 11	chr2:10,182,976-10,194,963
MODY8	*CEL*	Carboxyl Ester Lipase	chr9:135,937,365-135,947,248
MODY9	*PAX4*	Paired Box 4	chr7:127,250,346-127,255,982
MODY10	*INS*	Insulin	chr11:2,181,009-2,182,571
MODY11	*BLK*	BLK Proto-Oncogene, Src Family Tyrosine Kinase	chr8:11,351,510-11,422,113
MODY12	*ABCC8*	ATP-Binding Cassette Subfamily C Member 8	chr11:17,414,432-17,498,449
MODY13	*KCNJ11*	Potassium Inwardly Rectifying Channel Subfamily J Member 11	chr11:17,406,795-17,410,878
MODY14	*APPL1*	Adaptor Protein, Phosphotyrosine Interacting with PH Domain and Leucine Zipper 1	chr3:57,261,765-57,307,499

**Table 2:** The genetic variants identified in Western Siberia patients with a phenotype of maturity onset diabetes of the young (MODY).

Patient ID, Gender	Gene	Variant Status	Nucleotide Changes *	Amino Acid Changes	Location	Genotype	Minor Allele Frequency (gnomAD)	dbSNP ID	ClinVar Variation ID	HGMD	Pathogenicity According to ACMG [21], Evidence	LOVD Database ID	Segregation with Phenotype in Family
P6, Female	*GCK*	Known	c.106C>T	p.Arg36Trp	Exon 2	Heterozygous	0.000014	rs762263694	431973	CM940823	PR	GCK_000007	Yes
P50, Male						Heterozygous							Yes
P59, Female	*GCK*	Known	c.110T>C	p.Met37Thr	Exon 2	Heterozygous	NA	NA	NA	NA	PR	GCK_000100	Yes
P17, Female	*GCK*	Known	c.130G>A	p.Gly44Ser	Exon 2	Heterozygous	NA	rs267601516	76898	CM013265	PR	GCK_000029	Yes
P74, Female	*GCK*	Known	c.238G>A	p.Gly80Ser	Exon 3	Heterozygous	NA	rs1554335761	449415	CM970630	PR	NA	Yes
**P51, Male**	***GCK***	**Novel**	**c.424A>T**	p.Lys142*	**Exon 4**	**Heterozygous**	**NA**	**NA**	**NA**	**NA**	**Pathogenic, PVS1, PM2, PM1, PP1**	**NA**	**Yes**
**P4, Male**	***GCK***	**Novel**	**c.436C>G**	**p. Leu146Val**	**Exon 4**	**Heterozygous**	**NA**	**NA**	**NA**	**NA**	**Pathogenic, PS1, PS3, PM2, PP1**	**NA**	**Yes**
P10, Male	*GCK*	Known	c.449T>A	p. Phe150Tyr	Exon 4	Heterozygous	NA	rs193922297	129142	CM097114	PR	NA	Yes
**P86, Female**	***GCK***	**Novel**	**c.517_520del**	p.Ala173Glnfs*30	**Exon 5**	**Heterozygous**	**NA**	**NA**	**NA**	**NA**	**Pathogenic, PVS1, PS1, PM2, PM4, PP1, PP3**	**NA**	**Yes**
P90, Male	*GCK*	Known	c.517G>A	p.Ala173Thr	Exon 5	Heterozygous	NA	NA	NA	NA	Pathogenic, PS1, PM2, PM5, PP1, PP3	GCK_000217	Yes
**P80, Female**	***GCK***	**Novel**	**c.542T>A**	**Val181Asp**	**Exon 5**	**Heterozygous**	**NA**	**NA**	**NA**	**NA**	**Pathogenic, PS1, PM2, PM5, PP1, PP3**	**NA**	**Yes**
**P46, Female**						**Heterozygous**							**Yes**
P14, Female	*GCK*	Known	c.580-1G>A	-	Intron 5	Heterozygous	NA	rs1554335421	449414	CS052048	PR	NA	Yes
P30, Female	*GCK*	Known	c.660C>A	p.Сys220*	Exon 6	Heterozygous	NA	NA	NA	CM020443	PR	NA	Yes
P68, Female	*GCK*	Known	c.700T>C	p.Tyr234His	Exon 7	Heterozygous	NA	NA	NA	CM096864	PR	NA	Yes
P40, Female	*GCK*	Known	c.752T>C	p. Мet251Тhr	Exon 7	Heterozygous	NA	rs193922326	36251	CM096876	PR	NA	Yes
P67, Male	*GCK*	Known	c.755G>A	p.Сys252Tyr	Exon 7	Heterozygous	NA	NA	NA	CM021266	PR	NA	NA
P83, Male	*GCK*	Known	c.771G>A	p.Trp257*	Exon7	Heterozygous	NA	NA	NA	NA	Reported in [22]	NA	NA
P3, Male	*GCK*	Known	c.772G>T	p.Gly258Cys	Exon 7	Heterozygous	NA	rs1583596378	804857	CM032578	PR	NA	NA
P77, Female													Yes
**P88, Female**	***GCK***	**Novel**	**с.782G>C**	**p.Gly261Ala**	**Exon 7**	**Heterozygous**	**NA**	**NA**	**NA**	**NA**	**Pathogenic, PS1, PM2, PM5, PP3**	**NA**	**NA**
**P54, Male**	***GCK***	**Novel**	**c.864-1G>T**	**-**	**Intron 7**	**Heterozygous**	**NA**	**NA**	**NA**	**NA**	**Pathogenic, PVS1, PM2, PP1, PP3**	**NA**	**Yes**
**P57, Male**	***GCK***	**Novel**	**c.1113C>A**	p.Cys371*	**Exon 9**	**Heterozygous**	**NA**	**NA**	**NA**	**NA**	**Pathogenic, PVS1, PS1, PM1, PM2, PM5, PP1**	**NA**	**Yes**
P70, Female	*GCK*	Known	c.1120G>A	p.Val374Met	Exon 9	Heterozygous	NA	rs1415041911	447380	CM096927	PR	NA	Yes
P61, Female	*GCK*	Known	c.1148C>A	p.Ser383*	Exon 9	Heterozygous	0.0000042	rs777870079	NA	CM032579	PR	NA	Yes
**P87, Male**	***GCK***	**Novel**	**c.1327G>A**	**p.Glu443Lys**	**Exon 10**	**Heterozygous**	**NA**	**NA**	**NA**	**NA**	**Likely pathogenic, PM1, PM2, PP2, PP3**	**NA**	**NA**
**P19, Female**	***HNF1A***	**Novel**	**c.18C>G**	**p.Ser6Arg**	**Exon 1**	**Heterozygous**	**NA**	**NA**	**NA**	**NA**	**Likely pathogenic, PS4, PM2, PP1, PP2, PP3**	**NA**	**Yes**
P11, Female	*HNF1A*	Known	c.185A>G	p.Asn62Ser	Exon 1	Heterozygous	0.00012	rs377129682	447485	CM064300	PR	HNF1A_000235	NA
**P34, Female**	***HNF1A***	**Novel**	**c.526 +1G>T**	**-**	**Intron 2**	**Heterozygous**	**NA**	**NA**	**NA**	**NA**	**Pathogenic, PVS1, PM2, PP1, PP2, PP3**	**NA**	**Yes**
P65, Female	*HNF1A*	Known	c.608G>A	p.Arg203His	Exon 3	Heterozygous	0.000008	rs587780357	129235	CM993816	PR	HNF1A_000137	Yes
**P91, Female**	***HNF1A***	**Novel**	**c.713G>A**	**p.Arg238Lys**	**Exon 3**	**Heterozygous**	**NA**	**NA**	**NA**	**NA**	**Likely pathogenic, PS1, PM2, PP2, PP3**	**NA**	NA
**P78, Male**	***HNF1A***	**Novel**	**c.713 +2T>A**	**-**	**Intron 3**	**Heterozygous**	**NA**	**NA**	**NA**	**NA**	**Pathogenic, PVS1, PM2, PP1, PP2, PP3**	**NA**	**Yes**
P82, Female	*HNF1A*	Known	c.779C>T	p.Thr260Met	Exon 4	Heterozygous	0.0000040	rs886039544	265436	CM971457	PR	HNF1A_000148	NA
P16, Female	*HNF1A*	Known	c.1522G > A	p.Glu508Lys	Exon 6	Heterozygous	0.00044	rs483353044	135665	CM082841	PR	HNF1A_000214	NA
P81, Male	*HNF1A*	Known	c.1768G>A	p.Val590Met	Exon 9	Heterozygous	0.0000042	rs1168108747	447484	NA	Uncertain significance, PM2, PP1, PP2, PP3	NA	Yes
P73, Male	*HNF1A*	Known	c.160C>T	p.Arg54*	Exon 1	Heterozygous	0.0000042	rs766956862	805632	CM032035	PR	HNF1A_000220	Yes
	*ABCC8*	Known	c.1562G>A	p.Arg521Gln	Exon 10	Heterozygous	0.000095	rs368114790	157683	CM1212138	PR	ABCC8_000172	
P12, Male	*ABCC8*	Known	c.4369G>C	p.Ala1457Thr	Exon 36	Heterozygous	NA	rs72559717	NA	CM011260	PR	NA	Yes
P27, Female	*HNF1B*	Known	c.1006C>G	p.His336Asp	Exon 4	Heterozygous	0.0002	rs138986885	595653	CM067046	PR	HNF1B_000125	No

* RefSeq reference transcript: *GCK* (NM_000162.5), *ABCC8* (NM_000352.3), *HNF1B* (NM_000458.2), and *HNF1A* (NM_000545.6). HGMD: Human Genome Mutation Database; LOVD database: Leiden Open Variation Database; NA: not available; PR: previously reported to be associated with a pathological phenotype (in the literature, in LOVD, ClinVar, or HGMD) and was not classified according to ACMG recommendations [21]. Variants identified for the first time are boldfaced.

## Data Availability

The data presented in this study are available on request from the corresponding author. The data are not publicly available due to privacy.
